# Electromagnetic Surgical Navigation for Accurate Pediatric Tumor Localization: A Preclinical Animal Cadaver Study

**DOI:** 10.3390/cancers18172831

**Published:** 2026-09-01

**Authors:** Jasper M. van der Zee, Marijn A. J. Hiep, Vera J. van Boheemen, Guus M. J. Bökkerink, Wout J. Heerink, Matthijs Fitski, Michiel A. J. van de Sande, Stefanie Veraa, Marc H. W. A. Wijnen, Harald C. Groen, Alida F. W. van der Steeg

**Affiliations:** 1 Department of Pediatric Surgery, Princess Máxima Center for Pediatric Oncology, 3584 CS Utrecht, The Netherlandsv.j.vanboheemen@prinsesmaximacentrum.nl (V.J.v.B.);; 2 Department of Surgical Oncology, The Netherlands Cancer Institute, 1066 CX Amsterdam, The Netherlands; 3 Technical Medicine, University of Twente, 7522 NB Enschede, The Netherlands; 4 Department of Orthopedics, Leiden University Medical Center, 2333 ZG Leiden, The Netherlands; 5 Department of Clinical Sciences, Division of Diagnostic Imaging, Faculty of Veterinary Medicine, Utrecht University, 3584 CL Utrecht, The Netherlands

**Keywords:** tracked ultrasound, image-guided surgery, pediatric oncology, ultrasound-based registration, animal cadaver study

## Abstract

**Background**: Localization of non-palpable, non-visible, and difficult location tumors in children can be surgically complex. Incomplete resections upstage the tumor, leading to a significant reduction in survival and the intensification of adjuvant treatment, affecting quality of life. Surgical navigation set-ups showed promising results in adults but have not yet been validated in complex pediatric oncology surgery. In this study, an in-house developed electromagnetic surgical navigation set-up was evaluated in a pre-clinical cadaver study. **Methods**: Pediatric oncology surgeons (*n* = 8) performed navigated tumor resection in eight simulated surgeries. Embalmed piglets with tumors simulated through insertion underwent preoperative CT imaging for 3D modeling. Navigation was initialized with an ultrasound-based registration procedure. During the procedures, the surgeons had to localize and remove the simulated tumors using the navigation set-up, and the ease of use was evaluated by the system usability score (SUS). The registration and localization accuracies were calculated with the surface and target registration errors, respectively. **Results**: The registration was successful in 7/8 experiments. The median surface and target registration errors were <2 mm and <3 mm, respectively. The surgeons scored above average acceptance of the navigation set-up with a median SUS of 72 (IQR: 62–80). **Conclusions**: The navigation set-up was successful overall, with surgeons being satisfied concerning the usability and accuracy of the technique. This study supports progression toward clinical evaluation.

## Simple Summary

The surgical removal of pediatric tumors can be challenging if those appear to be incredibly small, not palpable, or located in difficult locations. In adults, recent improvements have resulted in innovative surgical navigation opportunities to aid the surgeon during surgical tumor localization. This set-up was redeveloped for use in children, which is evaluated on animal cadavers in this study. Participating pediatric surgeons used the set-up in a simulated setting, removing simulated tumors in embalmed piglets. The performance was registered, and the surgeons were asked about the user friendliness of the set-up. Based on the results, use of this set-up in pediatric surgical oncology is deemed possible. In the future, this navigation set-up may simplify challenging oncological surgeries in children, aiming to improve the surgical treatment of childhood cancer.

## 1. Introduction

Tumor localization in pediatric surgical oncology can be particularly challenging due to substantial biological changes induced by preoperative chemo- or radiotherapy, making tumors non-palpable or visually indistinct. In pediatric sarcoma surgery, complete tumor removal remains the cornerstone of treatment, as it is strongly associated with an increased survival rate [1]. Improvements in surgical 3D planning have already decreased the extensiveness of surgery. However, in specific cases, the intraoperative localization of the extent of the tumor can still be challenging, contributing to incomplete resection rates of more than 50%, such as in non-rhabdomyosarcoma soft tissue sarcomas [2]. In neuroblastoma, tumors may be strongly adherent to vital structures or encase major vessels, increasing the risk of vascular injuries during resection [3,4]. At the same time, preserving surrounding healthy tissue requires a high level of surgical expertise, given the close proximity of tumors to critical anatomical structures. Additionally, the localization of malignant lymph nodes (e.g., metastatic retroperitoneal lymph nodes after paratesticular rhabdomyosarcoma) or node sampling during a total nephrectomy can be challenging, as they may be extremely small and therefore easily overlooked [5,6]. Together, these factors underscore both the need for extensive surgical planning as well as an improved intraoperative localization. Currently, this relies primarily on the surgeon’s experience and the interpretation of stacked preoperative imaging slices. Surgeons translate these 2D slices mentally into 3D, which they use during surgery. However, as a result, the current methods may lead to suboptimal guidance toward the target and unnecessary damage to healthy tissue.

**Figure 1:**
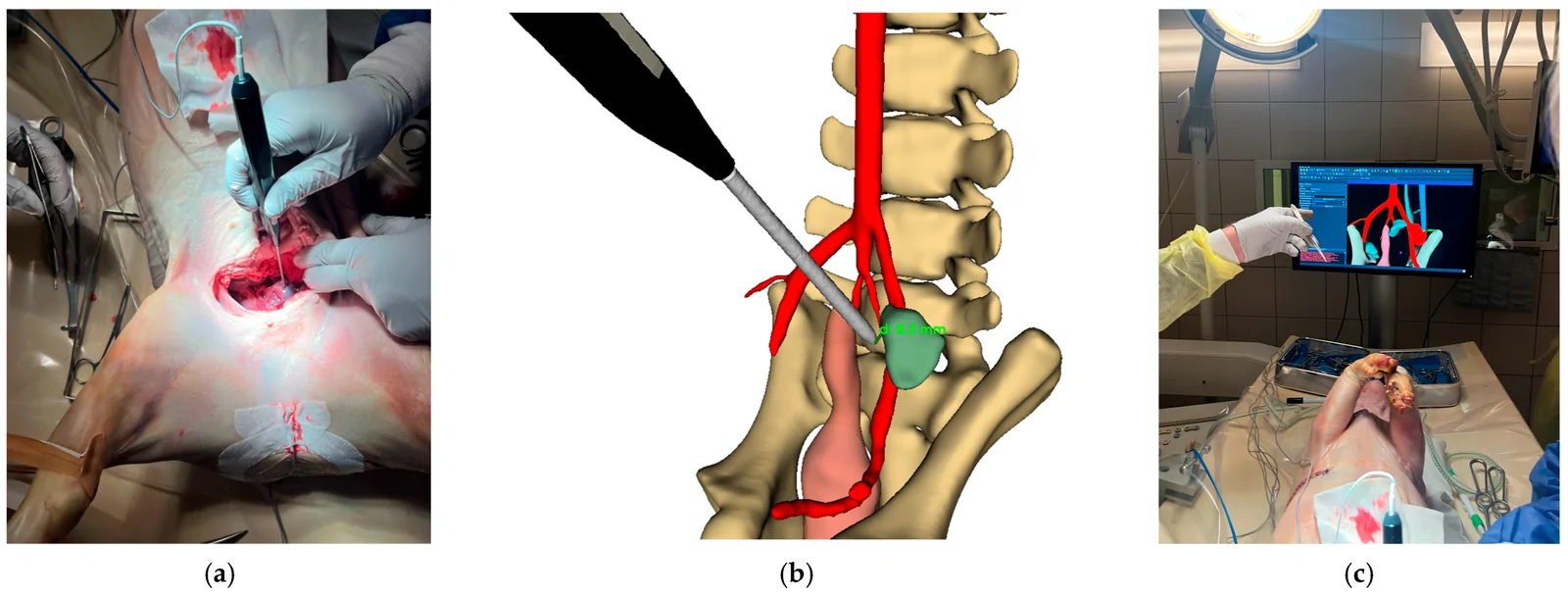
In-house developed surgical navigation set-up that aids localization for complex resection in a simulated animal cadaver setting. (**a**) Photograph of the actual situation in which the surgeon holds the tracked surgical pointer in the wound. (**b**) The intraoperative feedback of the navigation set-up, showing the anatomical 3D model with the tumor (green), bones (sand), artery (red), and surgical pointer with the shortest distance to the target. (**c**) The navigation is shown on a monitor that is positioned above the cadaver.

Advanced intraoperative guidance helps surgeons with the localization of malignant tissue while avoiding critical structures [7]. One promising intraoperative guidance approach is electromagnetic navigated surgery, in which a navigated surgical pointer provides real-time guidance, as shown in Figure 1. Integrating surgical navigation with 3D models and imaging may improve a surgeon’s spatial understanding, which may lead to more precise resections, less damage to healthy tissue, and less intraoperative complications [8].

The Netherlands Cancer Institute (Antoni van Leeuwenhoek hospital, Amsterdam, the Netherlands) studied the clinical relevance of electromagnetic surgical navigation during challenging adult oncologic surgery. This surgical research team developed and subsequently implemented electromagnetic surgical navigation to facilitate adequate resections during complex procedures, such as for locally recurrent rectal cancer procedures, malignant lymph node dissection, and liver surgeries [9,10]. This led to an increased success rate of the removal of metastatic retroperitoneal lymph nodes from 68% up to 93% [9]. Furthermore, the complete resection rate in adult patients with locally recurrent rectal cancer increased by 30% [8]. Moreover, the use of navigated surgery was investigated, and the participating surgeons reported that the electromagnetic navigation tool simplified complex surgery, facilitated tumor localization, and made surgeons feel more confident during surgery [8].

Although innovative surgical navigation tools have shown improvement in surgical accuracy, confidence, and even surgical outcomes for adult surgical oncology, the implementation in pediatric oncology surgery is lacking [11]. Pediatric oncologic surgery is not comparable to adult surgery in several ways: children have smaller body dimensions and relatively larger tumors, and the surgical field is narrower. Consequently, for technology translation to children, such specific challenges and differences must be addressed, focusing on younger children between 3 and 6 years. This has led to our in-house developed surgical navigation set-up tailored for use in children. Although the set-up passed pre-clinical phantom testing, further testing together with end users in a realistic surgical simulation is necessary before clinical implementation can occur [12].

In adults, human cadavers can be used to simulate clinical settings and to practice with new surgical set-ups [13]. Although such cadaver models are crucial during the development of novel set-ups, the availability of pediatric cadavers is extremely limited, and alternatives had to be explored [14]. Ultimately, embalmed animal cadavers showed their potential in real-life simulations to practice specific pediatric surgical approaches [15]. For our purpose, cadavers of young pigs—piglets—were chosen to simulate the body dimensions of children [16,17]. We presume that if the set-up is deemed surgically feasible for younger children, then this will also be the case for older children and adolescents. In this study, the feasibility of the set-up will be evaluated in a preclinical animal cadaver study together with the end users. If the cadaver study demonstrates the surgical feasibility, then we can continue with clinical trials in pediatric oncologic surgery.

## 2. Materials and Methods

### 2.1. Study Design

Eight participants performed the simulated surgeries: six pediatric surgeons (five consultants with an average of 18 years of experience (STD: 6 years) and one fellow with 6 months of experience) and two orthopedic pediatric surgeons (one consultant with 15 years of experience and one fellow with one year of experience). At the Surgical Oncology Department of the Princess Máxima Center (Utrecht, the Netherlands), complex pediatric tumor resections are usually performed by two pediatric surgeons. Therefore, the simulated procedures were performed by the same surgeons who routinely perform these specific surgical procedures in the clinical practice.

In total, eight simulated surgeries were performed in duos across different anatomical locations, as shown in Table 1. During each experiment, the surgeons were asked to perform a total resection of the simulated tumor conforming with the conventional surgical approach. Additionally, the surgical navigation set-up was available upon request to aid during tumor localization. The clinical requirement for the accuracy was predefined by pediatric oncology surgeons in our center for tumors fixated to the bone to be 4 mm; otherwise, it was 5 mm.

### 2.2. Navigation Set-Up

**Figure 2:**
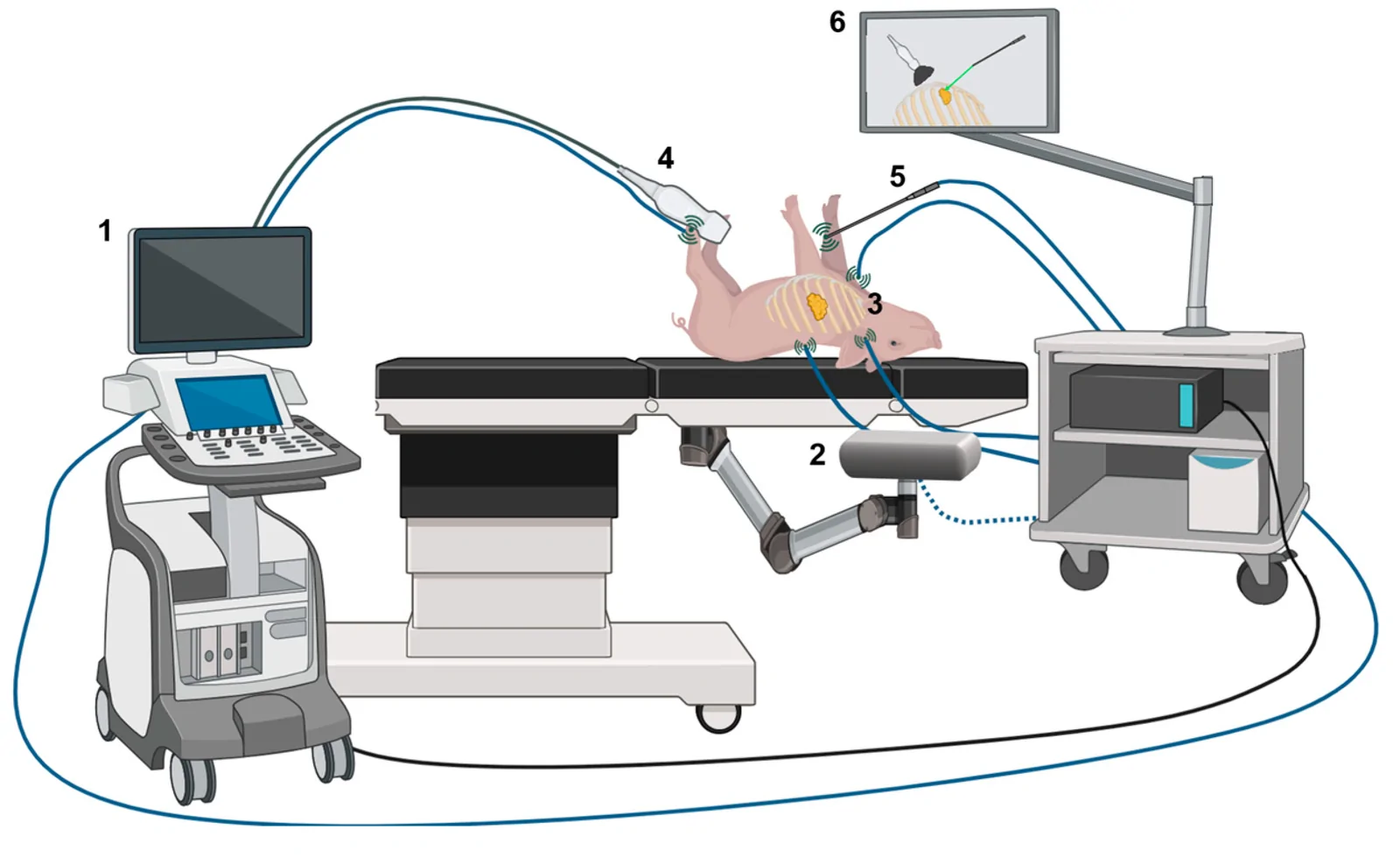
Schematic overview of the navigation set-up: (**1**) ultrasound device, (**2**) electromagnetic planar field generator, (**3**) electromagnetic patient sensors, (**4**) tracked ultrasound transducer with clip-on tool, (**5**) surgical pointer, and (**6**) monitor displaying navigation interface.

The intraoperative navigation set-up comprised different components: an electromagnetic tracking system (Aurora^®^ V3, Northern Digital Inc., Waterloo, ON, Canada), electromagnetic sensors (Ø2.5 mm × 12 mm long, Aurora^®^ 6DOF Tool sensor, Northern Digital Inc.), surgical pointer (Aurora^®^ 6DOF Probe, Northern Digital Inc.), Epiphan DVI2USB 3.0 frame grabber (Epiphan System Inc., Ottawa, QC, Canada), and a Hitachi Avius ultrasound imaging device (Hitachi Medical Systems, Tokyo, Japan) with both a convex (8–4 MHz) and a linear (14–6 MHz) transducer. The whole set-up was run on a computer workstation (Intel^®^ i7-1300h, GPU NVIDIA RTX 4080 Laptop, 32 GB Ram). A schematic overview of the whole set-up is shown in Figure 2. During the procedure, 3D Slicer (Version 5.8.1) navigation software was used, which is open-source software for 3D modeling, image processing, and computer science [18]. Streaming of the tracking and imaging data was performed via the PLUS Toolkit (version 2.9.0) [19].

**Figure 3:**
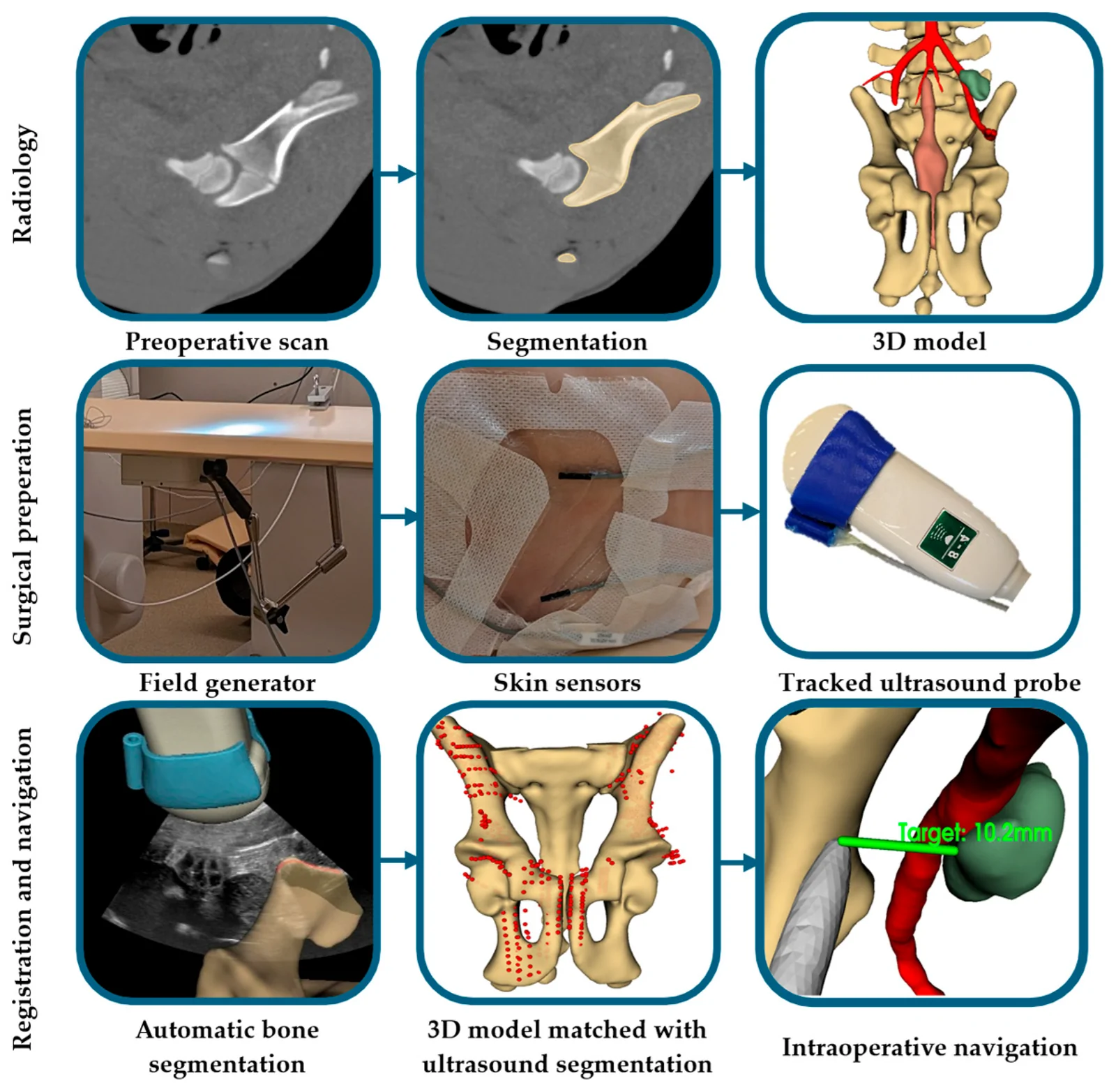
Surgical navigation workflow, including radiological and surgical preparation, the tracked ultrasound-based registration, and subsequent navigation. The tumor is shown in green, the clip-on-tool in blue, and the bone surface detected on ultrasound is indicated by the red line, resulting in the red point cloud.

Ultrasound imaging was acquired with a linear and a curved ultrasound transducer, depending on the simulated surgical case. For each probe, a 3D printed clip-on tool was developed and equipped with a sensor for tracking. Tracked ultrasound calibration was performed using the pointer-based method for both ultrasound probes [20]. Patient tracking occurred with sensors, and surgeons could use the surgical pointer. During the procedure, navigation was visualized on a monitor inside the operating room. The surgical navigation workflow using the pelvic bones is shown in Figure 3.

### 2.3. Porcine Cadaver Preparation and Radiology

Four piglets with body weights of ±15 kg and a median head-body length of 73.8 cm (72.3–74.3 cm) were obtained post-mortem from a commercial butcher, and as no live animals were involved, no ethical approval or animal experimentation license was required. The specimens were embalmed to improve their preservation, and this allowed reuse of the same animal for multiple experiments, thereby minimizing the number of animals used, in accordance with the principles of reduction [13]. Embalming was performed via a canula that was inserted into the common carotid artery for vascular access. The embalming fluids were sourced from the Dodge Company (Billerica, MA, USA), and the procedure was performed at the Department for Anatomy and Physiology at the Faculty for Veterinary Medicine (Utrecht, The Netherlands). For vascular visibility, a contrast mixture was inserted into the same cannula using a mixture of PEG200 and Omnipaque one minute before scanning to prevent fluid shifting, according to the protocol of Bruch et al. [21,22].

Tumors were simulated with a mixture consisting of demi water with 1.5 *w*/*w*% methylcellulose, 1.5 *w*/*w*% agarose, and 3.5 *w*/*w*% glycerol, following the protocol of Scott et al. [23]. When heated to 95 °C, the mixture dissolved, and 1.6 *w*/*w*% gelatin and 4 *w*/*w*% Omnipaque (300 mg I/mL) were added to improve adhesion to the surrounding tissue and obtain sufficient CT contrast. The simulated tumors were injected by a veterinary radiologist under ultrasound guidance, and those became stiff when cooled down below 35 °C. The tumors (±10–15 per cadaver) were injected according to the anatomical locations as described in Table 1 for every cadaver. More tumors were injected than strictly required to provide flexibility in selecting both the suitable cadaver and the most representative target for each experiment.

After injection, the cadavers were preoperatively CT-scanned (Siemens Somatom Definition AS 64-slice sliding gantry, Erlangen, Germany) at the Faculty of Veterinary Medicine in Utrecht, the Netherlands using a voxel size of 0.8 × 0.8 mm, slice thickness of 1 mm, and a standard b30f soft tissue reconstruction kernel. These preoperative scans were used to derive the 3D surgical planning using Mimics Innovation Suite (Materialise, version 19.0, Leuven, Belgium). A wide range of anatomical structures (e.g., bones, organs, tumors, and arteries) were segmented for each cadaver along with the simulated tumors. Subsequently, since all cadavers were prepared with simulated tumors, the cadaver with the most suitable tumor simulation was selected by the technical physician for each specific experiment.

### 2.4. Surgical Preparation

In the described surgical navigation set-up, the procedure started by matching the preoperative model with the intraoperative location of the cadaver, the so-called registration. An electromagnetic field generator was positioned underneath the surgical bed for real-time tracking, and the preoperative 3D planning would be matched with the cadaver position by the tracked intraoperative ultrasound. Animal tracking was performed using sensors, either placed on the animal skin (with plasters) or directly onto the tissue (using surgical glue or with sutures) or firmly fixated to the bone (using screws or Kirschner wires). The specific sensor position for each surgical case is described in Appendix A.

### 2.5. Registration and Navigation

During registration, the technical physician (JVDZ) handled the registration procedure in the software, and the surgeons performed the required ultrasound acquisitions. First, an initial registration was performed. Therefore, a single-point registration was used when the cadaver orientation on the surgical table matched the CT acquisition, assuming alignment of the coordinate axes. Otherwise, a three-point landmark-based registration was performed to compute the correct orientation. The landmarks were found on ultrasound, being bony, vessel, or other anatomical landmarks.

Second, an ultrasound-based bone registration was performed based on either the surface of the bones or the kidney, as shown in Table 1. In the case of the bone-based registration, the bone surface was imaged, and a 3D point cloud was computed via real-time surface detection with our predefined UNeXt model for the bone surface [24]. For the kidney-based registration, the technical physician manually selected surface points on the ultrasound image. The resulting point cloud was then matched to the corresponding preoperative 3D model of the bones or organs. Registration was considered successful when two predefined criteria were met: (1) a surface distance of ≤5 mm between the point cloud and the 3D model and (2) visual confirmation of the overlap of the 3D model with the ultrasound by the technical physician performing the registration. False positives were considered predicted surface points on a distance >5 mm from the surface, and these points were removed.

After registration, the surgical pointer was visualized relative to the preoperative derived 3D models in the navigation interface and the surgical procedure started. To illustrate, the procedure for the simulated rib tumor, the nephroblastoma and the lymph node dissection are shown in the video (Supplementary Materials Videos S1–S3).

### 2.6. Study Measurements

The accuracy of the registration was measured using the surface registration error (SRE) in millimeters between the 3D model and the registered detected bone surface points on ultrasound. Registration was considered successful if the superimposed bone models were visually aligned with the bone surface on the ultrasound. To define the surgical accuracy upon the target tumor, the surgeons were asked to localize the tumor with guidance of the navigation set-up using the surgical pointer. Once located, they were asked to expose the simulated tumor and place the surgical pointer on top of the surface to save datapoints. The distance error between the actual tumor surface points and the closest surface point in the 3D model was defined as the target registration error (TRE). For the muscular tumor, the tumor surface was probed using a different cadaver postoperatively by the technical physician, conforming with standard surgical procedure, and this tumor was not exposed during the surgery.

Each procedure was evaluated by the two participating surgeons in terms of technical performance, user-friendliness, and satisfaction using a questionnaire for each participant. In the first part of the questionnaire, the system usability score (SUS) was defined by scoring 10 statements on a five-point Likert scale (1 = totally disagree, 2 = disagree, 3 = neutral, 4 = agree, and 5 = totally agree), which was subsequently converted to a 0–100 score [25]. Higher SUSs are correlated with greater user-friendliness of the set-up, and a threshold of >70 means the set-up is feasible and may be acceptable for clinical use. In the second part, the surgeons had to score different statements that compared the use of navigation with the conventional, no-navigation situation on a five-point Likert scale. For each specific statement, a score above three indicated a preference for the use of navigation. Lastly, the surgeons had to answer different open questions.

### 2.7. Statistics

All statistical analyses were performed using descriptive statistics. Because the number of experiments varied per anatomical location, no formal normality testing was conducted. Accuracy was assessed by calculating the median and interquartile range (IQR) of the SRE and TRE values for each experiment. Given the differences in the number of data points across the anatomical locations and tasks, accuracy data were not pooled across experiments, locations, or tasks. Instead, results were reported and evaluated at the individual experiment level.

For the usability assessment, two surgeons completed a questionnaire after each successful experiment, defined as successful registration followed by tumor localization. In the event of registration failure, tumor localization could not be performed, and no usability questionnaire was administered. Such cases were excluded from the usability analysis. As surgeons were selected from a pool of eight participants, some participated in multiple experiments. Therefore the usability scores were not fully independent across the experiments. For each experiment, the scores of the two surgeons were averaged to obtain a consensus score per experiment. The overall usability outcome was then summarized across the successful experiments using the median and IQR.

## 3. Results

Ultrasound-based registration was successful in 7/8 experiments. For the successful registrations, the median SRE values between the 3D point cloud and the registered surfaces (i.e., bone and organ) was less than 2 mm. During the registration process, we achieved a sufficient visual overlay between the ultrasound imaging and the 3D models after approximately 70% of the available surface was scanned.

**Figure 4:**
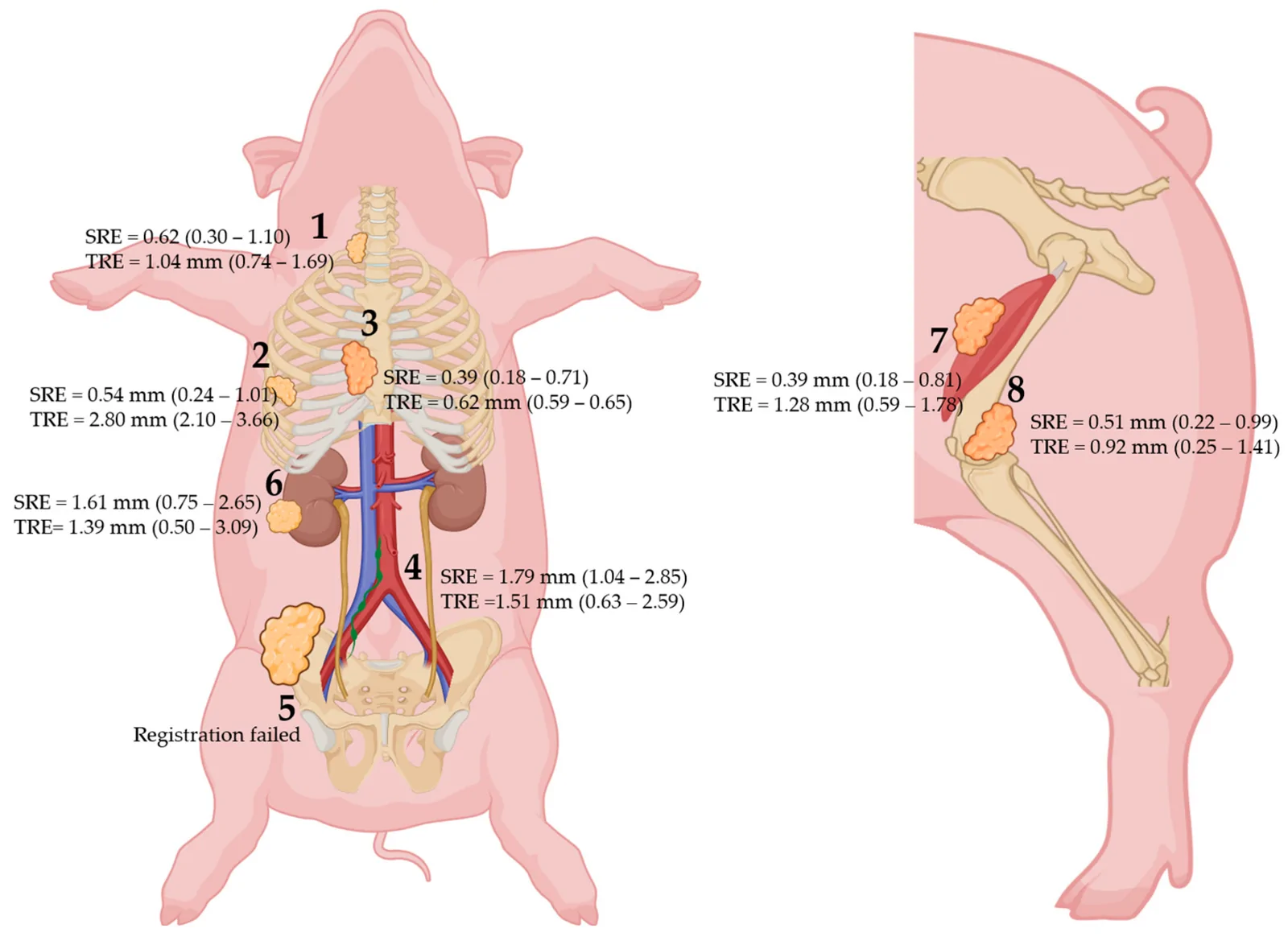
The median registration accuracy (SRE) and surgical accuracy (TRE), with the interquartile ranges, for the eight simulated surgeries. Bone (sand), kidney (brown), artery (red), vein (blue), ureter (yellow), muscle (red) and tumor (orange).

The registration failed for the pelvic bone experiment with the tumor located in the os ilium and the piglet positioned on its side, as the localization of anatomical landmarks was difficult. This resulted in an inaccurate three-point initial registration and a failed definitive registration. The accuracy results for all individual cases are shown in Figure 4. Tumor localization resulted in a median TRE value <3 mm. The lowest and highest TRE values were observed in the sternal case (0.62 mm, IQR: 0.59–0.65) and kidney case (2.80 mm, IQR: 2.10–3.66), respectively. The supplementary document provides box plots of the registration and localization accuracies for the individual experiments.

The seven successful experiments resulted in 14 completed questionnaires, yielding a median consensus SUS of 72 (IQR: 62–80). The predefined threshold for good clinical acceptance (SUS ≥ 70) was reached in five of the seven procedures. In general, the surgeons found the surgical navigation helpful during localization of the tumor and lymph nodes, potentially making procedures easier. Furthermore, they found the set-up to be of added value during every experiment, except for the nephroblastoma experiment. For this experiment, only one of the two surgeons found the navigation to have added value.

During localization, the surgical pointer was the preferred tracking tool above the tracked ultrasound probe. Compared with the conventional approach, surgeons scored five out of six statements in favor of the use of the surgical navigation set-up, as shown in Table 2. Although the proposed set-up has shown clinical potential, no surgeon was convinced regarding the effect upon patient survival (median: 3.0 [3.0–3.0]), as this remains a multi-variable outcome measure. Finally, the surgeons commented positively on the realistic tactile feeling of the embalmed piglets and their representative body dimensions compared with a young child, as well as the preservation over time.

## 4. Discussion

In this study, we evaluated an in-house developed surgical navigation set-up with different simulated surgeries using embalmed piglets, simulating the pediatric oncology patient group. We found the set-up to be accurate according to our predefined clinical requirements, and the participating surgeons scored the set-up to be clinically acceptable and had a preference toward the navigation after seven successful procedures.

### 4.1. Simulated Surgical Setting

We aimed for the best surgical representation for pediatric surgeries using embalmed piglet cadavers with injected simulated tumors [17,26]. The protocol used for these simulated tumors was straightforward and provided a realistic surgical scenario. Similar findings were reported by Székely et al. [27], who used the same protocol for tumor simulation in lung tissue for thoracic surgery training. Based on both their and our experiences, the simulated targets closely mimicked small non-palpable targets, had good CT appearance, and were realistic for the simulated surgical case. Although the tumors were realistic, the simulated nephroblastoma was insufficient, as these lesions were already visible on the surface of the kidney and could be excised with minimal dissection. Nevertheless, this tumor simulation method enabled testing of a wide variety of cases to evaluate the system’s performance in localizing both bone-fixated and soft tissue tumors.

Pediatric surgeons are familiar with human anatomy, but identifying anatomical landmarks in piglets can be difficult due to the anatomical differences between these animals and humans [28]. As a result, the localization of known bony landmarks required for the initial registration was challenging. Specifically, the identification process of such landmarks failed for the pelvic bone tumor experiment, which led to failed registration, as a correct initial alignment is essential [29].

### 4.2. Registration Performance and Surgical Accuracy

The registration accuracies found in this study are comparable to those of commercial surgical navigation systems [30]. Moreover, although direct comparison is limited, since the cadaver model did not simulate physiological or intraoperative motions, such as respiration, cardiac motion, or table movements, the localization performance observed in this study was comparable or appeared to be more accurate than the accuracies reported in adult surgical oncology, where accuracies of 3–4 mm for pelvic tumors [31], 2.6 mm for pelvic lymph nodes [29], and 3.2 mm for liver tumors [10] have been reported. Therefore, the localization accuracy in clinical pediatric practice may be lower than that observed in the present cadaver study. Furthermore, accuracy measurements are inherently challenging, and the TRE acquisition in this study was not fully homogeneous across all experiments, particularly for the muscular case. Therefore, direct comparisons of TRE values should be interpreted with caution.

Automatic bone segmentation performed sufficiently, and no significant outliers were found by the algorithm. Therefore, the algorithm appeared to perform robustly, even though piglets were imaged instead of children, as the algorithm was trained using pediatric imaging data only [24]. The registration of the femur, sternum, and kidney was shown to be straightforward, resulting in high accuracies comparable to the results of the phantom study of Van Boheemen et al. [12]. Therefore, the registration and localization accuracy are sufficient compared to the required clinical accuracies as defined by our center. However, the registration on the ribs was challenging due to its moon-shaped cylinders, resulting in high registration uncertainty along the length of the rib. Comparable to the ribs, Fanti et al. described that for registration on a diaphysis, the lack of unique morphological features could result in inaccuracies [32]. To overcome this, we observed that the landmarks used during the initial registration should involve the transition between the cartilage and the rib to define the starting point of the rib. Subsequently, the ultrasound sweep should now follow the ribs to prevent the registration shifting, resulting in a successful registration. Based on the available literature and the best of our knowledge, this is the first study to report ultrasound-based rib registration.

### 4.3. Advances in Soft Tissue Surgical Navigation

In the field of pediatric surgical oncology, the localization of small or non-palpable tumors in a relatively small surgical working field can be particularly difficult compared with adult surgery. Although existing surgical navigation techniques are useful to guide surgeons to such challenging targets, these techniques are focused mostly on the localization of bone tumors. Those commercial set-ups, manufactured by companies such as BrainLab or Medtronic, use optical tracking systems (OTSs). In a review by Sorriento et al. [30], OTSs were considered to be superior in terms of accuracy compared with electromagnetic tracking systems (EMTSs). However, these systems require relatively large reference bodies and a line of sight with the tracker. Therefore, OTSs are the gold standard for navigation if a reference body can be rigidly fixated to the body. For soft tissue tracking, such reference bodies cannot be fixated, limiting the possibility for localization of soft tissue tumors rising from the thoracic wall, abdomen, or pelvic areas [33]. To overcome this, Smit et al. [34] and Nijkamp et al. [35] proposed a method with small EMTS sensors directly attached to either the liver or the skin, highlighting the potential of EMTS-based navigation setups for real-time tracking of mobile soft tissue targets and supporting the use of skin or organ-attached sensors for accurate localization. This approach formed the fundamental basis for patient and target tracking in the navigation set-up used in this study. Therewith, this set-up is unique, and together with tracked ultrasound-based registration methods, its opens the possibility to use navigated pediatric surgery for more challenging targets, such as retroperitoneal pelvic lymph nodes.

For pediatric oncology surgery, a set-up meeting the specific requirements for soft tissue navigation did not exist. The in-house developed set-up of the Princess Maxima Center is considered to be the first approach that enables real-time tracking of mobile soft tissue targets. The performance of this set-up was examined in the benchmark study by van Boheemen et al. [12], which confirmed its performance in a controlled setting. With the results of the present cadaver study, the achieved accuracy is comparable to this benchmark, and the advancement for the ability of tracking soft tissues was also deemed sufficient. With the development of our set-up, a transition from navigation from static rigid anatomy toward dynamic soft tissue-based navigation is now available for pediatric surgical oncology.

To further improve the user experience, specifically for organ tracking, the currently used tracking system (NDI Aurora^®^) lacks wireless sensors. The surgeons observed that little cable tension occasionally displaced the sensor from the kidney surface, resulting in reduced navigation accuracy. This likely affected the SUS in these specific cases. The use of wireless sensors could potentially overcome this issue, making navigation more accurate, robust, and user-friendly [36]. Advances in sensor technology from traditional inductive micro-sensors, which are used in the current tracking system, toward magneto-sensitive sensors could enable new concepts in wireless sensor designs [37].

### 4.4. Clinical Implementation

During the resection of the muscle tumor around the femur, some technical difficulties occurred. Although the procedure itself proceeded according to the planned workflow, these issues influenced the subjective experience of one surgeon, who rated the procedure with a lower SUS. The other surgeon did not perceive the technical difficulties as disruptive, illustrating that the perceived flow of the procedure can vary substantially between individuals.

During the experiments, the surgeons were satisfied with the ease of use of the set-up and commented positively, with statements such as “With the surgical navigation setup being congruent with my own intraoperative perception, my surgical confidence is increased bidirectionally”, and “The setup will make invisible and non-palpable tumors visible”. This satisfaction was also reflected in the questionnaire results. Although the current study did not evaluate clinical outcomes, surgeons perceived the set-up as having the potential to facilitate complex procedures and improve the surgeon’s confidence. The reported usability scores indicated acceptable usability, and surgeons described the navigation as intuitive. Mezger et al. [38] reported that surgical navigation enhanced the overall intraoperative spatial awareness and thereby increased surgeon confidence during complex procedures, which was underlined by the participating surgeons in our study.

A learning curve associated with the set-up is likely driven primarily by the technical physician, who is responsible for the technical set-up and operation, whereas the surgeon’s interaction with the navigation is known to be straightforward and expected to have a limited learning effect [39]. Nevertheless, a learning effect cannot be excluded, and both the technical physician and the surgeons had prior phantom-based experience. In clinical practice, adequate training and continuous technical support remain essential.

Specifically, the participating surgeons reported increased confidence during localization of either the tumor or the surrounding anatomy. Whether this translates into a reduction in surgical complications remains to be evaluated in future clinical studies. To illustrate this, navigation could be helpful to avoid vascular injury in the case of vascular encasement of the tumor. In future clinical settings, the set-up may aid in lymph node localization in metastatic disease or lymph node sampling after neuroblastoma and Wilms tumor resection. Moreover, the unique advantage of the small sensors is that real-time tracking of organs is now possible. Therefore, the investigated set-up demonstrated the feasibility of a navigation approach that may be applicable to a wide variety of pediatric tumors.

### 4.5. Limitations

This study has several limitations that should be acknowledged. First, as this was a single-center feasibility study, the experiments were evaluated individually, and the results could not be pooled to assess overall performance. Furthermore, the TRE measurements were not fully homogeneous across the experiments, and for the usability results, the same surgeon could be attributed to multiple experiments. Second, case selection was prone to selection bias as multiple tumors were simulated in different cadavers, and the technical physician selected the most suitable target for each experiment. Third, since the proposed set-up is designed for mobile soft tissues, the absence of breathing and cardiac motion represents a limitation inherent to the cadaver design. In addition, although the embalming process prolonged tissue preservation, tissue degradation over time still affected the tissue properties and odor. Finally, although electromagnetic field measurements were not performed to assess potential interference, the used materials were selected for their ferromagnetic properties. While the results of this preclinical feasibility study are promising, clinical performance must be further investigated in a clinical study.

## 5. Conclusions

The surgical usability of our in-house developed surgical navigation set-up tailored for use in pediatric oncology patients and tested in porcine cadavers was, overall, deemed feasible by the participating surgeons. The participating surgeons were satisfied with this set-up across different simulated pediatric oncology surgeries. Future studies should include clinical trials to ensure efficient translation of the current results in actual pediatric oncology surgery.

## Figures and Tables

**Table 1:** The eight simulated surgeries for each different anatomical region, target type, sensor locations for patient tracking, required accuracy, and surface structure used during the registration. Sensor placement differed between cutaneous and rigid fixation, as this was determined by the target and the expected tumor-skin motion. Tumors freely moving from the skin required direct fixation of the sensor to the tissue.

Case	Anatomical Region	Simulated Target	Sensor Location During Tumor Localization	Required Accuracy	Surface Used During Registration
1	Neck	Soft tissue tumor	Cutaneous (3x)	5 mm	Rib 1
2	Thorax	Tumor in the ribs	Cutaneous (3x)	5 mm	Rib IV-VII
3	Thorax	Sternal tumor	Cutaneous (3x)	4 mm	Sternum
4	Pelvic	Common iliac lymph nodes	Cutaneous (3x)	5 mm	Pelvis
5	Pelvic	Bone tumor in os ilium	Kirschner wire fixation with a 3D printed adapter (1x)	4 mm	Pelvis
6	Abdomen	Nephroblastoma (left and right)	Organ surface (1x)	4 mm	Kidney
7	Lower extremity	Muscle tumor in musculus femoris	Kirschner wire fixation with a 3D printed adapter (1x) and subsequently sutured to the muscle (1x)	4 mm	Femur
8	Lower extremity	Bone tumor in femur	Kirschner wire fixation with a 3D printed adapter (1x)	5 mm	Femur

**Table 2:** Preference of the used surgical navigation set-up compared with the non-navigation conventional method. On a scale of 1–5, a score lower than three indicates a preference for the conventional method, and a score higher than three indicates a preference to use the surgical navigation set-up for this surgical procedure (median and IQR [25th–75th]).

Efficiency of the Surgical Procedure upon:	Score:
Survival	3.0 [3.0–3.0]
Complications	4.0 [3.0–4.8]
Resection margins	4.0 [3.0–4.0]
Total time	4.0 [3.0–4.0]
Removal of the disease	4.0 [3.3–4.0]
Improved confidence in decisions and surgical handling	5.0 [3.3–5.0]

**Figure A1:**
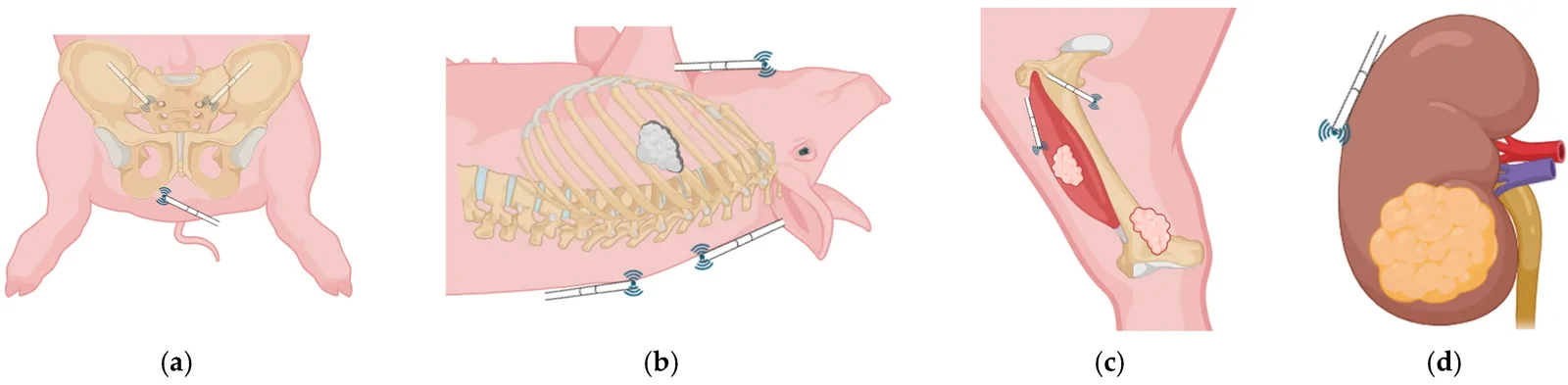
(**a**,**b**) Fixation of the electromagnetic sensors on the skin for tumors located in the pelvis or the thorax and (**c**) directly to the bone for tumors located in the bone and attached to the surface of an organ in the case of tumors located inside organs, such as muscles (**c**) or kidneys (**d**). Sensors (white), bone (sand), kidney (brown), artery (red), vein (blue), ureter (yellow), muscle (red) and tumor (either in grey, pink or yellow).

## Data Availability

The raw data supporting the conclusions of this article will be made available by the authors on request.

## Supplementary Materials

The following supporting information can be downloaded at https://www.mdpi.com/article/10.3390/cancers18172831/s1. Video S1: Rib. Video S2: Nephroblastoma. Video S3: LND. Figure S1: Boxplots of the registration accuracies across the different experiments. Figure S2: Boxplots of the localization accuracies across the different experiments.

## Abbreviations

The following abbreviations are used in this manuscript:

CT	Computed tomography
EM	Electromagnetic
MM	Millimeter
MR	Magnetic resonance
NDI	Northern Digital Incorporated
STD	Standard deviation
SUS	System usability score
TRE	Target registration error
2D	Two-dimensional
3D	Three-dimensional

## Appendix A

Comparable to the actual surgical procedure, the piglet was either firmly fixated using straps to prevent movement or not fixated if movement was required during surgery (e.g., orthopedic cases). The best method for sensor positioning and fixation was iteratively tested and evaluated. The most efficient sensor locations specified per tumor location and tracking requirements are shown in Figure A1.

### Appendix A.1. Tumors in the Neck, Chest Wall, Sternum, and Pelvis

Sensor fixation of two sensors on the back and one on the front of the piglet was considered to be most effective. In particular, during the registration of the pelvis (piglet laying on its side) or the ribs, a rolling movement of the piglet was observed, and therefore, one contra-lateral sensor was able to correct for these movements.

### Appendix A.2. Lower-Extremity Tumors

With tumors either in the bone or in the surroundings, sensors attached to the skin were able to correctly track movements before incision and during the earlier phases of the resection. As large incisions were required, and the bone or tumor was able to move independent of the skin, the sensor had to be fixated in a more rigid manner. Therefore, two options for sensor location were explored: either directly fixated to the bone for a bone tumor or attached to the muscle. In the first option, the bone was accurately tracked during all required surgical manipulations applicable in case the tumor was fixated to the bone (e.g., osteosarcomas). If the tumor was located in a large muscle around the bone (e.g., rhabdomyosarcomas), then a single sensor fixated directly on the muscle was able to track the muscle during surgical manipulation.

### Appendix A.3. Kidney Tumors

Fixation of the sensor on the kidney surface was challenging due to a relatively limited space and adhesion issues with the surgical glue. Therefore, once the sensor was fixated, the sensor and cable had to be carefully approached during manipulation of the resection.
