# Supplementary material for: Electromagnetic Surgical Navigation for Accurate Pediatric Tumor Localization: A Preclinical Animal Cadaver Study
Source: Cancers (Basel). 2026 Sep 1;18(17):2831. doi: 10.3390/cancers18172831 (PMC13565530; doi:10.3390/cancers18172831)
Supplement: Supplementary file 1 [file cancers-18-02831-s001.zip › Supplementary material.pdf]

Supplementary materials of the paper entitled: *Electromagnetic Surgical Navigation for Accurate Pediatric Tumor Localization: A Preclinical Animal Cadaver Study*

Jasper M. van der Zee 1, Marijn A. J. Hiep 2, Vera J. van Boheemen 1,3, Guus M. J. Bökkerink 1, Wout J. Heerink 2, Matthijs Fitski 1, Michiel A. J. van de Sande 1,4, Stefanie Veraa 5, Marc H. W. A. Wijnen 1, Harald C. Groen 2 and Alida F. W. van der Steeg 1,*

1 Department of Pediatric Surgery, Princess Máxima Center for Pediatric Oncology, 3584 CS Utrecht, The Netherlands
2 Department of Surgical Oncology, The Netherlands Cancer Institute, 1066 CX Amsterdam, The Netherlands
3 Technical Medicine, University of Twente, 7522 NB Enschede, The Netherlands
4 Department of Orthopedics, Leiden University Medical Center, 2333 ZG Leiden, The Netherlands
5 Department of Clinical Sciences, Division of Diagnostic Imaging, Faculty of Veterinary Medicine, Utrecht University, 3584 CL Utrecht, The Netherlands
* Correspondence: a.f.w.vandersteeg@prinsesmaximacentrum.nl

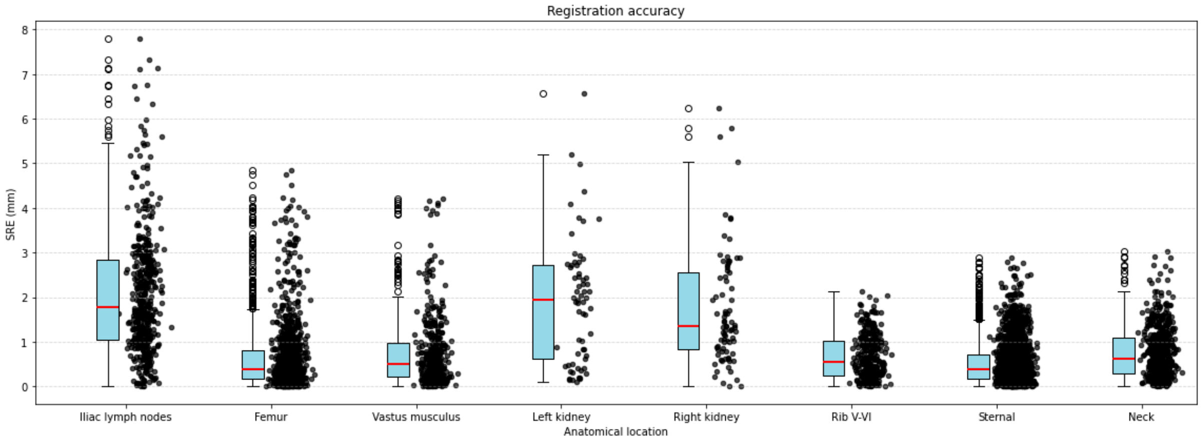

Figure S1: Boxplots of the registration accuracies across the different experiments.

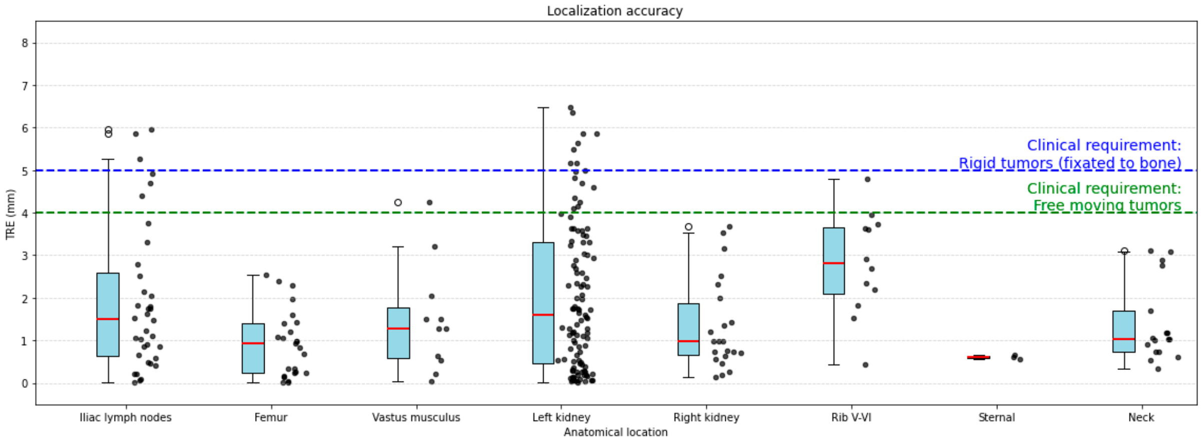

Figure S2: Boxplots of the localization accuracies across the different experiments.
